# Comparison of Composition, Free-Radical-Scavenging Capacity, and Antibiosis of Fresh and Dry Leave Aqueous Extract from *Michelia shiluensis*

**DOI:** 10.3390/molecules28165935

**Published:** 2023-08-08

**Authors:** Wentao Wu, Gaoyu Li, Weijuan Zhou, Enbo Wang, Xia Zhao, Xiqiang Song, Ying Zhao

**Affiliations:** Hainan Key Laboratory of Biology of Tropical Flowers and Trees Resources, Forestry Institute, Hainan University, Haikou 570228, China; wuwentao2021@163.com (W.W.); ligaoyu0816@163.com (G.L.); zhou2677842315@163.com (W.Z.); baroque2014@outlook.com (E.W.); zhaopray@163.com (X.Z.); songstrong@hainanu.edu.cn (X.S.)

**Keywords:** *Michelia shiluensis*, antibiosis, free-radical-scavenging capacity, gas chromatography–mass spectrometry

## Abstract

Numerous plants of medicinal value grow on Hainan Island (China). Given the lack of knowledge on the phytochemical and pharmacological properties of *Michelia shiluensis* Chun and Y. F. Wu (*M. shiluensis*), the application of natural antioxidants and antimicrobials in the food industry has attracted increasing interest. This study aimed to compare the chemical composition, free-radical-scavenging capacity, and antibiosis of aqueous extracts of the fresh and dried leaves of *M. shiluensis.* The aqueous extract of the leaves of *M. shiluensis* was obtained using steam distillation, and its chemical components were separated and identified via gas chromatography–mass spectrometry (GC-MS). The free-radical-scavenging capacity and antibiosis were determined. Further, 28 and 20 compounds were isolated from the fresh leaf aqueous extract of *M. shiluensis* (MSFLAE) and dried leaf aqueous extract of *M. shiluensis* (MSDLAE), respectively. The free-radical-scavenging capacity of MSFLAE and MSDLAE was determined by the 2,2-diphenyl-1 picrylhydrazyl (DPPH) method, which was 43.43% and 38.74%, respectively. The scavenging capacity of MSFLAE and MSDLAE determined by the 2,2′-azino-bis (3-ethylbenzothiazoline-6-sulfonate (ABTS)) method was 46.90% and 25.99%, respectively. The iron ion reduction capacity of MSFLAE and MSDLAE was determined by the ferric-reducing antioxidant power (FRAP) method as 94.7 and 62.9 μmol Fe^2^⁺/L, respectively. This indicated that the two leaf aqueous extracts had a certain free-radical-scavenging capacity, and the capacity of MSFLAE was higher than that of MSDLAE. The antibiosis of the two leaf aqueous extracts on the three foodborne pathogenic bacteria was low, but the antimicrobial effects on Gram-positive bacteria were better than those on Gram-negative bacteria. The antibiosis of MSFLAE on *Escherichia coli* and *Staphylococcus aureus* was greater than that of MSDLAE. Finally, MSFLAE and MSDLAE both had certain free-radical-scavenging capacities and antibiosis, confirming that the use of this plant in the research and development of natural antioxidants and antibacterial agents was reasonable. Plant aqueous extracts are an essential source of related phytochemistry and have immense pharmacological potential.

## 1. Introduction

*M. shiluensis* is an evergreen tree found in the tropics. It belongs to the family Magnoliaceae. The genus *Michelia* comprises approximately 80 species and is widely distributed in tropical and subtropical South and Southeast Asia. It is widely used in landscaping and is suitable for the environment in many parts of the world. *Michelia* is considered an essential group of ornamental and medicinal plants globally. It contains various natural products, including sesquiterpene lactones, alkaloids, terpenes, flavonoids, and other polyphenols, and has significant pharmacological effects [1,2]. Additionally, *Michelia* has antioxidant, antibacterial, and anti-inflammatory activities [3,4,5]. Its traditional use against diseases such as fever, leprosy, gout, and inflammation has been documented in China and India [6,7].

Plant-derived bioactive ingredients are known for their higher bioavailability and fewer side effects. In recent years, plant extracts have been widely used as antimicrobial and antioxidant agents for pharmaceutical, cosmetic, and food applications. Medicinal plant-derived therapies have been shown to be extremely safe and effective for treating recalcitrant bacterial infections. The urgent need for natural antimicrobial agents arises from the ineffectiveness of conventional antibiotics in treating infections caused by drug-resistant bacteria [7]. Many bacterial infections are closely associated with oxidative stress induced by free radicals, which are an important part of aerobic life and metabolism. Scavenging reactive oxygen species represents one of the possible modes of action of antioxidants [8]. Therefore, the free-radical-scavenging properties of plant extracts are of great significance for preventing and treating cancer and other diseases.

At present, several studies exist on the active properties of extracts from plants belonging to the Magnoliaceae family. For example, the extract of *M. shiluensis* contains 16 sesquiterpenoids, of which lipiferolide has obvious cytotoxicity to human cancer cells [9]. Similarly, *M. compressa var. lanyuensis* extract contains compounds such as liriodenine and β-sitostenone, which significantly inhibit melanin synthesis [10]. Polysaccharides with pyran groups in *Magnolia kwangsiensis* extract have significant antitumor effects on human lung cancer cells [11]. However, previous studies on the biological activities and active components of the genus *Michelia* have mainly focused on a few species. Hence, research on other species of the genus *Michelia* is lacking. Considering the increasing popularity of natural medicinal plants and the significant biological activities of *Michelia*, this study was novel in analyzing the chemical composition, free-radical-scavenging ability, and antibacterial effect of its leaf extracts. The results of this study provided new ideas for using *Michelia* plant resources in functional food and medical applications.

## 2. Results and Discussion

### 2.1. GC-MS Component Analysis of MSFLAE and MSDLAE

**Figure 1:**
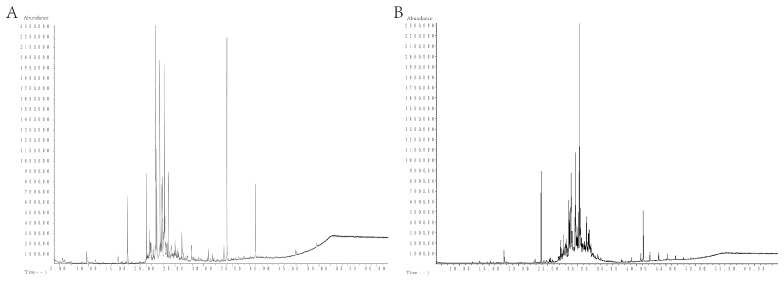
Total ion flow diagram of the components of MSFLAE and MSDLAE. (**A**) MSFLAE; (**B**) MSDLAE.

**Figure 2:**
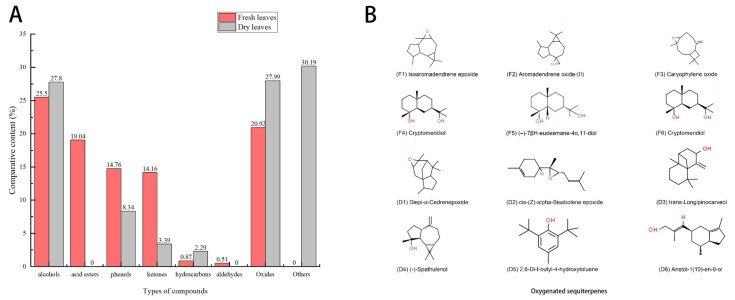
Analysis of compositions in MSLFAE and MSDLAEE. (**A**) Types and contents of compounds; (**B**) major sesquiterpene constituents.

Table 1 shows 28 components isolated and identified from MSFLAE, accounting for 95.76% of the relative content (Figure 1A). MSFLAE mainly comprised alcohols, ketones, aldehydes, esters, hydrocarbons, and oxides, of which the relative content of five alcohols was 25.5%. The relative content of oxides, acid esters, phenols, and ketones was 20.92%, 19.04%, 14.76%, and 14.16%, respectively. The relative content of hydrocarbons and aldehydes was 0.87% and 0.51%, respectively (Figure 2A). The sesquiterpene alcohols with a higher content were cryptomeridiol (9.67%) and (-)-7βH-eudesmane-4α, 11-diol (11.66%). The compound with the highest ketone content was 1,2,3,6-tetramethyl-bicyclo [2.2.2] octa-2,5-diene (7.2%). The compounds with higher oxide content were isoaromadendrene oxide (8.86%), aryophyllene oxide (2.95%), and aromadendrene oxide (II) (5.26%). The compound with the highest acid content was 3-methyl-2-butenoic acid, tridec-2-ynyl ester (11.29%). The content of 2,2′-methylenebis [6-(1,1-dimethylethyl)-4-methylphenol] reached 10.30%. Phenolic compounds are substances with anticancer, immune system support, antibacterial, antiviral, and antifungal properties, which protect the skin from ultraviolet radiation [12]. Caryophyllene oxide and isoaromadendrene oxide are oxygenated terpenoids tested in vitro as antifungal agents against dermatophytes and have good antibacterial effects [13,14]. Volatile oils containing these two chemical components also have free-radical-scavenging capabilities [15]. MSFLAE has a high content of sesquiterpene alcohols and sesquiterpene oxides, such as isoaromadendrene oxide, cryptomeridiol, (-)-7βH-eudesmane-4α, 11-diol, and other components. Terpenoids and many of their oxygenated derivatives are considered critical raw materials for the pharmaceutical, cosmetic, and food industries due to their strong biological activity and aromatic properties [16,17,18,19]. Studies conducted by Sahoo et al. [20], Cheng et al. [21], Chen et al. [22], and Dai et al. [23] supported these findings, demonstrating a prevalence of sesquiterpenes and their derivatives, such as sesquiterpene alcohols and sesquiterpene oxides, within the *Michelia* genus. Other substances, including monoterpene alcohols, β-caryophyllene, and β-elemene, are also notably present. Furthermore, diterpenes and homoterpenes are rarely observed in MSFLAE and other *Michelia* species, possibly because the members of the Magnoliaceae family are the most primitive angiosperms and, therefore, lack or have lower levels of homoterpenes [22].

Further, 42 components were isolated from MSDLAE (Table 1), of which 20 components were identified, accounting for 69.81% of the relative content (Figure 1B). MSDLAE mainly comprised alcohols, ketones, phenols, hydrocarbons, and oxides (Figure 2A). Of the 20 identified constituents, 8 were terpene alcohols, making up 27.8% of the relative content, and 3 were oxides, accounting for 27.99% of the relative content. Phenolic compounds, hydrocarbons, and ketones represented 8.34%, 2.29%, and 3.39% of the relative content, respectively. The relative content of hydrocarbons and ketones was relatively low. The higher alcohols were cryptomeridiol (8.06%) and (-)-spathulenol (6.63%). The compounds with the highest oxide content were diepi-α-cedrenepoxide (20.01%), β-cedrenoxide (4.22%), and *cis*-Z-α-bisabolene epoxide (3.76%). The compound with the highest phenolic content was 2,6-di-*tert*-butyl-4-methyl phenol (5.15%). Studies found that (-)-spathulenol inhibited the growth of *S. aureus,* was cytotoxic to KB cells, and moderately inhibited human topoisomerase I [24]. Cryptomeridiol, an antispasmodic active component of Cymbopogon proximus [25], was also present in *Geranium macrorrhizum* and *Cassia tora* [26,27]. Many sesquiterpene lactones were chemically distinct from other group members by lactone structure and had a wide range of prominent biological activities, including antitumor and cytotoxic effects [28,29]. MSDLAE was also rich in sesquiterpene components, including diepi-α-cedrene epoxide, cryptomeridiol, and *cis*-Z-α-bisabolene epoxide, among others (Figure 2B). Venkatadri et al. [30] found *cis*-Z-α-bisabolene epoxide and phenol, 2,4-bis (1,1-dimethylethyl) as the main components in the bark of *Michelia nilagirica*. Additionally, the decline and disappearance of acid ester components in MSDLAE might be due to the hydrolysis of lipids into alcohols and carboxyl compounds under long-term heating and drying.

Significant differences were found in the chemical composition of MSFLAE and MDLAE. MSDLAE contained more compounds, and the relative content of compounds also changed. In general, numerous compounds revealed an increasing trend. Some high-boiling-point compounds were retained, while others were lost or transformed. This was due to the evaporation or conversion of leaf components into new compounds during the drying process, resulting from heat, enzymatic action, oxidation, and other factors [31]. Only four components were shared between MSFLAE and MSDLAE: cryptomeridiol, *cis*-Z-α-bisabolene epoxide, (-)-spathulenol, and 2,6-di-tert-butyl-4-methylphenol. After drying, the content of these compounds fluctuated, increasing and decreasing to varying extents. The relative contents of alcohols and oxides in the dried aqueous extract increased by 3% and 7.07%, respectively. Sesquiterpene alcohols and sesquiterpene oxides, intrinsic to both MSFLAE and MSDLAE, were the characteristic components and served as crucial indicators distinguishing them from the components in other plant species.

### 2.2. Antioxidant Activity

**Figure 3:**
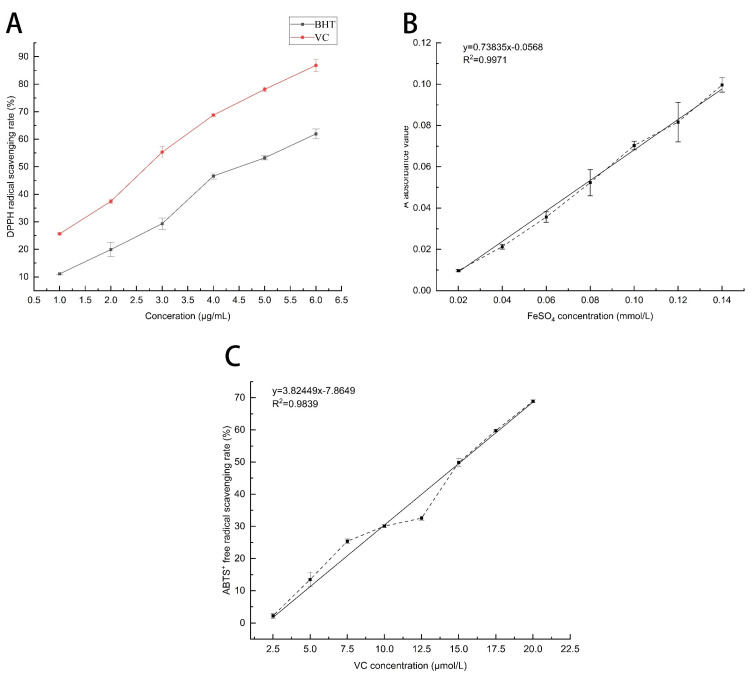
Free-radical-scavenging capacity of MSFLAE and MSDLAE. (**A**) Effect of different concentrations of BHT and VC on the clearance rate; (**B**) the standard curve of FeSO_4_; (**C**) and the standard curve of sodium ascorbate.

DPPH is a stable organic free radical widely used to evaluate the antioxidant capacity of compounds in plants by quenching the stable purple DPPH to yellow with a spectrophotometer [32]. The principle of antioxidant activity is based on the ability to neutralize free radicals by electrons, and phytochemicals such as pentacyclic triterpenoids have been recorded to have this effect [33]. The free-radical-scavenging capacity of DPPH gradually increases with increasing VC and BHT concentrations (Figure 3A). The standard curve equations for VC and BHT were defined as Y = 13.84X + 11.80 (*R*^2^ = 0.996) and Y = 10.63X + 0.58 (*R*^2^ = 0.997), respectively. The DPPH free-radical-scavenging capacity of MSFLAE was recorded at 43.43%, equivalent to 2.28 μg/mL VC and 4.1 μg/mL BHT, respectively. The DPPH free-radical-scavenging capacity of MSDLAE was measured at 38.74%, equivalent to 1.95 μg/mL of VC and 3.7 μg/mL of BHT, respectively. The antioxidant activity exhibited by MSFLAE and MSDLAE does not significantly deviate from that observed in other medicinal plants and herbs [34,35]. At the same concentration, the free-radical-scavenging capacity of MSFLAE was significantly higher than that of MSDLAE, which is consistent with the finding of Singh et al. [36]; fresh sample extracts have higher antioxidant potential. Kessy et al. [37] reported that the radical-scavenging capacity of FRAP and DPPH of dried *Litchi Pericarp* peel extracts decreased significantly by 52.53% and 25.55%, respectively, which corresponds to the high degradation of bioactive phenolic compounds. In addition, many plant aqueous extracts contain high levels of sesquiterpene components, which have similar antioxidant activity to phenolic compounds, disrupting free radical chain reactions and causing their irreversible oxidation to inert compounds [38].

The ferric iron reduction antioxidant potential assay is useful for evaluating the antioxidant activity of MSFLAE and MSDLAE. The method is based on the reduction of a colorless iron complex (Fe^3+^-tripyridyltriazine) to a blue ferrous complex (Fe^2+^-tripyridyltriazine) by the antioxidant action of the donor at low PH [39]. As illustrated in Figure 3B, the standard curve equation is defined as Y = 0.7835X − 0.00568 (*R*^2^ = 0.997). Based on this standard curve, the iron reduction capacities of MSFLAE and MSDLAE are quantified as 94.7 and 62.1 μmol Fe^2^⁺/L, respectively. The iron reduction capacity of MSFLAE surpasses that of MSDLAE, an observation in agreement with the DPPH measurements. Aldogman et al. [40] conducted a study on the biological activity of *Mentha suaveolens* leaf volatile oil and discovered that the antioxidant capacity of fresh leaf volatile oil (94.50%) was also superior to that of the dry leaf volatile oil (90%). This was attributed to the high content of rosmarinic acid, ferulic acid, and other sesquiterpene phenolic compounds present in the fresh leaf volatile oil. 

The free-radical-scavenging capacities of MSFLAE and MSDLAE were evaluated using the ABTS method. The standard curve for VC was established as Y = 3.2844X − 17.8464 (*R*^2^ = 0.98). The free-radical-scavenging capacities of MSFLAE and MSDLAE were 46.90% and 25.99%, respectively, which is equivalent to 19.71 and 13.34 μmol/L of VC, respectively. The IC_50_ of VC was found to be 20.65 μmol/L. When compared to VC, both MSFLAE and MSDLAE demonstrated a weaker free-radical-scavenging capacity. This is consistent with previous findings. Aazza et al. [41] reported similar antioxidant activity (282.6 μmol/L) on aqueous extract of *Salvia miltiorrhiza*. The free-radical-scavenging ability of MSFLAE and MSDLAE was measured using DPPH, ABTS, and FRAP methods. The results show that MSFLAE has a higher free-radical-scavenging capacity than MSDLAE, which may be due to the fact that MSFLAE contains more sesquiterpene alcohols and oxides. However, the free-radical-scavenging capacity of MSFLAE and MSDLAE is not significantly high, which can be attributed to the lower concentration of chemical constituents in their aqueous extracts. Essentially, they only encompass the water-soluble fraction of the respective essential oils. Consequently, while their biological activity is lower, so is their toxicity level, rendering them simpler to apply [42].

### 2.3. Determination of Antibacterial Activity

**Figure 4:**
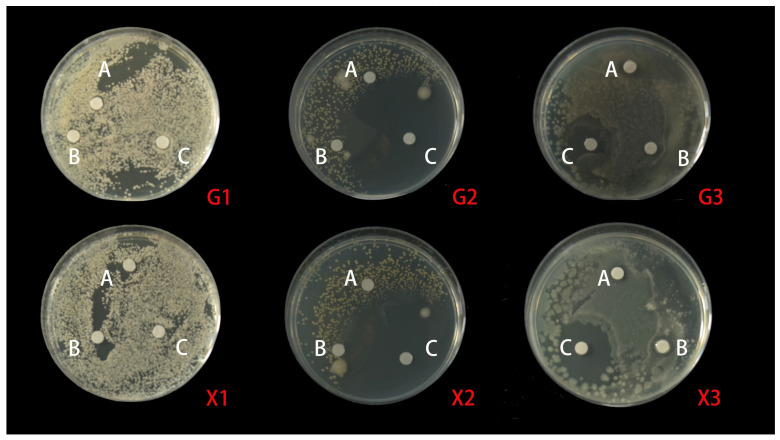
A schematic diagram of the antibacterial circle of MSFLAE and MSDLAE on the three tested bacteria. (A) Aqueous extract; (B) Tween-80; (C) penicillin; (1) *C. albicans*; (2) *S.aureus*; (3) *E. coli*; (X1–3) MSFLAE; (G1–3) and MSDLAE.

In order to study the antimicrobial potential of MSFLAE and MSDLAE, three foodborne pathogens, *E. coli* (Gram-negative bacteria) and *S. aureus* (Gram-positive bacteria), as well as the fungus *C. albicans*, were selected. The results revealed variable antimicrobial activities of MSFLAE and MSDLAE against these foodborne pathogens, with MSFLAE showing slightly superior inhibition against *E. coli* and *C. albicans* compared to MSDLAE (Figure 4). The antimicrobial circle of MSFLAE against *E. coli*, *S. aureus*, and *C. albicans* was 8.44 ± 0.10, 8.71 ± 1.08, and 7.41 ± 0.19 mm, respectively. The antimicrobial circle of MSDLAE against *E. coli*, *S. aureus*, and *C. albicans* was 7.11 ± 0.89, 8.10 ± 1.36, and 8.40 ± 0.36 mm, respectively. MSFLAE and MSDLAE were less susceptible to Gram-negative bacteria than Gram-positive bacteria, which may be due to the presence of an outer membrane in the cell membrane of Gram-negative bacteria, which can act as a barrier to reduce or prevent the penetration of various antimicrobial drugs [43]. There was no significant difference in the antimicrobial effect of the MSFLAE and MSDLAE against the three foodborne pathogens, and both showed weak antibiosis. The antimicrobial activity of aqueous extract may be related to the high content of oxygenated sesquiterpenes and sesquiterpene alcohols in aqueous extracts [44] because the active sesquiterpenes belong to the guaiane and elemene-type skeletons, and these compounds all contain hydroxyisopropyl. Wu et al. [45] documented that guaiacol, along with four other sesquiterpenes isolated from *Michelia formosana*, exhibited significant antibacterial effects against wood rot bacteria. Conversely, Feng et al. [46] reported that the four monoterpenoids with a seven-membered ring system, isolated from the flower buds of *Magnolia biondii*, did not demonstrate antibacterial activity within the tested range. *E. coli* and *S. aureus* were extremely sensitive to PGSS, with inhibitory zones of 21.2 ± 0.34 and 49.78 ± 0.99 mm, respectively. The antibacterial effect of PGSS was significantly higher than that of MSFLAE and MSDLAE (Table 2). There were significant differences in the antibacterial effect of aqueous extracts of different plants. Ali Shtayeh et al. [47] reported that the aqueous extracts of *Phagnalon rupestre* and *Micromeria nervosa* showed significant antibacterial effect against *C. albicans*. However, Huseyin et al. [48] reported that aqueous extracts of *Citrus aurantium* flowers did not exhibit antibacterial activity against the tested bacteria in the concentration range used.

## 3. Materials and Methods

### 3.1. Chemicals and Reagents

2,4,6-tripyridyl-s-trizine (TPTZ), 2,2′-azino-bis (3-ethylbenzothiazoline-6-sulfonic acid (ABTS), 2,2′- azobis [2-methylpropanamidine] dihydrochloride (AAPH), 1,1-diphenyl-2-picrylhydrazyl (DPPH), sodium ascorbate (VC), and Tween-80: Sabouraud Dextrose Aga reagents were purchased from Solarbio (Beijing, China). Sodium acetate (trihydrate), ferrous sulfide (hexahydrate), glacial acetic acid, and 2,6-di-tert-butyl-p-cresol (BHT) were purchased from Xilong Science Co., Ltd. (Shenzhen, China). Penicillin G sodium salt (PGSS) was purchased from Zhongkeruitai Biotechnology Co., Ltd. (Beijing, China); *Escherichia coli* (ATCC25922; *E. coli*), *Staphylococcus aureus* (ATCC27217; *S. aureus*), and *Candida albicans* (ATCC10231; *C. albicans*) were purchased from Jiangsu Edison Biotechnology Co., Ltd. (Yancheng, China). Luria–Bertani culture medium was prepared by ourselves.

### 3.2. Plant Materials and Extraction

*M. shiluensis* leaves were collected from Haikou City, Hainan Province, in October 2021 (20° N, 110° E). Dr. Zhao Ying from the School of Forestry at Hainan University confirmed the source plant as *M. shiluensis*. The voucher specimens were stored in the herbarium of Danzhou Campus of Hainan University (SCUTA 2965). Aqueous extracts were derived using a previously established procedure [49] with minor alterations. MSFLAE was extracted via steam distillation. Furthermore, 500 g fresh leaves was connected to the distillation device for heating for 120 min according to the solid–liquid ratio of 1:7. Finally, the steam-condensed distillate was collected, and the upper trace of essential oil was separated to isolate MSFLAE, which was then used for the detection of biological activity. The preparation of MSDLAE was consistent with the aforementioned conditions except for drying to constant weight at 60 °C. Additionally, 500 mL of the aqueous extract was taken and concentrated to 10 ml using rotary evaporation for the determination of components through gas chromatography–mass spectrometry (GC-MS).

### 3.3. Chemical Analysis

The GC-MS test/experiment was conducted following the procedure given below: a HP-5MS 5% phenyl methyl siloxane (30 m × 0.25 mm × 0.25 μm) elastic quartz capillary column was used; the heating program was: column temperature 50 °C, 5 °C/min to 310 °C, keeping 10 min; gasification chamber temperature of 250 °C; the carrier gas was high purity He (99.999%); precolumn pressure of 43 kPa, carrier gas (He) flow rate of 1.0 mL/min; the injection volume was 1.0 μL; and the solvent delay time was 4 min. The mass spectrometry detection conditions were as follows: electron bombardment (EI) was used as the ion source, with an electron energy of 70 eV, interface temperature of 280 °C, ion source temperature of 230 °C, four-bar temperature of 150 °C, the tuning mode was standard tuning, electron multiplier voltage of 1718 kV, and mass scanning range of 40–800 m/z. By using the Data Analysis chemical workstation and following the above chemical analysis methods, combined with the Nist2005 and Wiley275 mass spectrometry libraries, and drawing on the identification methods of previous researchers and our research group, the detected chromatographic peaks were retrieved and manually analyzed with reference to the relevant literature.

### 3.4. Assay of DPPH Radical-Scavenging Activity

The DPPH assay was performed by a previously described method [50] with some modifications: 0.1 mmol/L DPPH solution was prepared by dissolving 5.91 mg of DPPH in 150 mL of 75% ethanol and the mixture was left to react in the dark for 30 min for further use. The 1 mg/mL VC and BHT solutions were diluted to different concentrations as control. Further, 2 mL of the sample was poured into a centrifugation tube and 1 mL of DPPH solution was added to it; the mixture was then left to react in the dark for 30 min. The absorbance of the reaction solution was measured at 517 nm and 75% ethanol was used as blank control. The DPPH free-radical-scavenging rate was calculated based on the formula used in previous studies.

$$DPPH\;Scavenging\;rate(\%)=\left(\frac{A_{0}-A_{1}}{A_{0}}\right)$$

### 3.5. Assay of FRAP Radical-Scavenging Activity

The FRAP assay was performed based on a previous procedure [51] with minor modifications. The FRAP working solution was prepared as follows: we took 10 mmol/L TPTZ solution with a solvent of 40 mmol/L hydrochloric acid (HCl) solution, 300 mmol/L sodium acetate buffer, and 20 mmol/L FeCl_3_ solution. The three solutions were mixed in a ratio of 1:10:1 and the mixture was used immediately. Further, 27.8 mg FeSO_4_·7H_2_O was diluted to 10 mL with deionized water at a concentration of 100 mmol/L and then diluted to different concentrations. Subsequently, 1 mL of FRAP working solution was made to react with 2 mL of different concentrations of FeSO_4_ solution, and the absorbance A value of the solution after the reaction was measured. The standard curve of FeSO_4_ was drawn using the mixed solution of ethanol and FRAP working solution as blank control. Finally, the absorbance of the solution after the reaction of the sample with the FRAP working solution was measured and substituted into the regression equation of the standard curve, which was repeated three times.

### 3.6. Assay of ABTS Radical-Scavenging Activity

The ABTS assay was performed based on the method proposed by Thaipong et al. [52] with some modifications. First, the acetic acid buffer with pH value of 4.3 was prepared; 0.075 mmol/L ABTS and 2 mmol/L AAPH solutions were mixed with this buffer. This mixture was then reacted in a 45 °C water bath for 60 min. The 1 mol/L VC solution was prepared as a control and diluted to different concentration gradients. Further, 2 mL of the mixture was mixed with 1 mL of the sample solution, and the absorbance value was determined at 734 nm. The standard curve was drawn with VC solution as the standard reference and deionized water as the blank control. The standard curve was prepared based on the following formula:

$${ABTS}^{+}\;Scavenging\;rate\left(\%\right)=\left(1-\frac{A_{0}}{A_{1}}\right)\times 100\%$$

where *A*_0_ was 1 mL of the sample solution added with 2 mL of mixed solution and *A*_1_ was 1 mL of deionized water added with 2 mL of the mixed solution.

### 3.7. Preparation of Bacterial Suspension and Drug Solution

*Escherichia coli* (ATCC25922), *Staphylococcus aureus* (ATCC27217), and *Candida albicans* (ATCC10231) were inoculated into the corresponding liquid media. The bacteria were cultured at 120 rpm and 37 °C for 24–48 h, and the fungi were cultured at 120 rpm and 25 °C for 48–72 h. Finally, the bacterial suspension was diluted to 10^6^–10^7^ CFU/mL, and 1 mg/mL PGSS solution and 1 mg/mL Tween-80 solution were used as controls.

### 3.8. Disk Diffusion Method

The antibacterial effect of hydrosol was determined by paper diffusion method [53,54]. Under aseptic conditions, 200 μL of the bacterial suspension used in the experiment was uniformly coated on the surface of the corresponding solid medium to prepare a bacterial plate. Three circular filter papers with a diameter of 6 mm were uniformly adsorbed on the bacterial plate. A total of 10 μL of aqueous extract, Tween-80, and PGSS were added to different filter papers. The bacteria were cultured at 37 °C for 24 h and the fungi were cultured at 25 °C for 48 h. Then, the diameter of their antimicrobial circle was determined.

### 3.9. Statistical Analysis

Data were presented as mean and standard error (SE). Data were submitted to one-way analysis of variance (ANOVA) with a comparison of means using Tukey’s test (*p* <  0.05), using IBM SPSS Statistics 26.0 software, Mapping using Origin 2021.

## 4. Conclusions

In this study, 28 and 42 different compounds were isolated from MSFLAE and MSDLAE, respectively. Significant differences existed in the components of the two, with only four common components. The dried extract had more kinds of compounds, and the relative content also changed. Sesquiterpene alcohols and sesquiterpene oxides were the main components in the extracts of the two aqueous solutions. The total content remained relatively stable after drying, and the aforementioned compounds contributed significantly to free-radical-scavenging ability and antibacterial effect. The free-radical-scavenging ability and trivalent iron-reducing ability of MSFLAE were slightly higher than those of MSDLAE, and both had certain antioxidant activity. No significant differences were observed in the antimicrobial activity of MSFLAE and MSDLAE against the three foodborne pathogenic bacteria, exhibiting approximately the same antimicrobial capacity and lower antimicrobial activity and higher susceptibility to Gram-positive bacteria. The aqueous plant extracts have high pharmacological potential and great application potential. Therefore, *M. shiluensis* can also be used as a potential source of functional food and medicinal antimicrobials. Moreover, further studies on its toxicity, in vivo efficacy, and mechanism of action are needed to elucidate its potential use.

## Figures and Tables

**Table 1:** Metabolic components of MSFLAE and MSDLAE.

Numbering	Compound	Retention Time/min		Molecular Formula	Comparative Content/%	
		MSFLAE	MSDLAE		MSFLAE	MSDLAE
1	3-methyl-Pentanal	5.505		C_6_H_12_O	0.51	
2	(*E*)-3-(2-hydroxyphenyl)-2-Propenoic acid	9.678		C_9_H_8_O_3_	1.63	
3	5-Methyl-2,4-diisopropylphenol	15.144		C_13_H_20_O	0.48	
4	2,6-Ditert-butyl-4-methyl phenol	16.806	23.857	C_15_H_24_O	3.05	5.15
5	β-Ionone	20.047		C_13_H_20_O	3.38	
6	*cis*-Z-α-bisabolene epoxide	20.759	30.149	C_15_H_24_O	1.54	3.76
7	(-)-Spathulenol	21.228	28.552	C_15_H_24_O	0.73	6.63
8	4-(2,2,6-Trimethyl-7-oxabicyclo [4.1.0]hept-4-en-1-yl)pent-3-en-2-one	21.56		C_14_H_20_O_2_	0.6	
9	3-Methyl-2-butenoic acid, tridec-2-ynyl ester	21.667		C_18_H_30_O_2_	11.29	
10	Isoaromadendrene epoxide	21.733		C_15_H_24_O	8.86	
11	Caryophylene oxide	21.863		C_15_H_24_O	2.95	
12	3a,9-Dimethyldodecahydrocyclohepta[d]inden-3-one	22.291		C_16_H_26_O	0.51	
13	Cryptomeridiol	22.391	29.703	C_15_H_28_O_2_	9.67	8.86
14	4-(1,5-Dihydroxy-2,6,6-trimethylcyclohex-2-enyl)bμt-3-en-2-one	22.552		C_13_H_20_O_3_	0.98	
15	1,2,3,6-tetramethyl-Bicyclo [2.2.2]octa-2,5-diene	22.659		C_12_H_18_	7.2	
16	Aromadendrene oxide-(II)	22.807		C_15_H_24_O	5.26	
17	Kessane	22.86		C_15_H_26_O	1.55	
18	valenca-1(10),8-dien-11-ol	23.116		C_15_H_24_O	2.2	
19	(–)-7βH-eudesmane-4α,11-diol	23.187		C_15_H_28_O_2_	11.66	
20	(*E*)-Atlantone	23.276		C_15_H_22_O	2	
21	5-epi-7α,15-dihydroxyacorenol	24.386		C_15_H_26_O_2_	1.24	
22	Ledene oxide-(II)	25.039		C_15_H_24_O	0.76	
23	1,2-Benzenedicarboxylic acid, bis(2-methoxyethyl) ester	26.214		C_14_H_18_O_6_	1.65	
24	Hexadecane	27.08		C_16_H_34_	0.36	
25	Cyclooctenone, dimer	27.852		C_16_H_24_O_2_	0.84	
26	Acetic acid, octadecyl ester	30.808		C_20_H_40_O_2_	0.64	
27	2,2′-methylenebis [6-(1,1-dimethylethyl)-4-methyl-Phenol	34.025		C_23_H_32_O_2_	10.39	
28	1,3-Benzenedicarboxylic acid, bis(2-ethylhexyl) ester	38.957		C_24_H_38_O_4_	3.83	
29	8-Hydroxy-2-octanone		17.554	C_8_H_16_O_2_		1
30	−		27.163			1.01
31	−		27.3			0.59
32	−		27.573			0.6
33	6-Isopropenyl-4,8a-dimethyl-1,2,3,5,6,7,8,8a-octahydronaphthalene-2,3-diol		27.709	C_15_H_24_O_2_		1.71
34	−		27.78			0.51
35	5,6,6-Trimethyl-5-(3-oxobut-1-enyl)-1-oxaspiro [2.5] octan-4-one		27.964	C_14_H_20_O_3_		0.96
36	−		28.03			0.87
37	−		28.095			1.21
38	4,4,11,11-tetramethyl-7-Tetracyclo [6.2.1.0(3.8)0(3.9)] undecanol		28.629	C_15_H_24_O		1.77
39	−		28.712			3.24
40	Aristol-1(10)-en-9-ol		28.867	C_15_H_24_O		3.91
41	−		28.932			8.98
42	−		29.14			0.92
43	−		29.282			0.52
44	6-(p-Tolyl)-2-methyl-2-heptenol		29.603	C_15_H_22_O		2.39
45	Diepi-α-Cedrenepoxide		30.356	C_15_H_24_O		20.01
46	β-Cedrenoxide		30.481	C_15_H_24_O		4.22
47	−		30.618			0.54
48	−		30.701			0.95
49	Spatulenol		30.837	C_15_H_24_O		0.51
50	−		30.974			1.13
51	−		31.235			0.86
52	trans-Longipinocarveol		31.532	C_15_H_24_O		2.82
53	−		31.763			0.7
54	11-hydroxy-valenc-1 (l0)-en-2-one		31.87	C_15_H_24_O_2_		1.43
55	−		31.994			1.48
56	−		32.291			0.98
57	−		32.535			0.5
58	−		33.461			0.52
59	−		33.983			0.44
60	Tetracosane		40.749	C_24_H_50_		0.54
61	2,2′-methylenebis [6-(1,1-dimethylethyl)-4-methyl-Phenol]		41.159	C_23_H_32_O_2_		3.19
62	Heneicosane		42.298	C_21_H_44_		0.59
63	Hexacosane		43.788	C_26_H_54_		0.66
64	Tricosane		45.218	C_23_H_48_		0.5

Note: “−” absent.

**Table 2:** Antibacterial effect of MSFLAE and MSDLAE.

Bacteria	Antimicrobial Circle (mm)			
	MSDLAE	MSFLAE	Tween-80	PGSS
*Escherichia coli*	7.11 ± 0.89 ^b^	8.44 ± 0.10 ^b^	7.57 ± 0.61 ^b^	21.2 ± 0.34 ^a^
*Candida albicans*	8.10 ± 1.36 ^a^	8.71 ± 1.08 ^a^	7.25 ± 0.06 ^a^	7.48 ± 0.04 ^a^
*Staphylococcus aureus*	8.40 ± 0.36 ^b^	7.41 ± 0.19 ^b^	8.23 ± 0.15 ^b^	49.78 ± 0.99 ^a^

Note: mean ± estimated SEM, *n* = 3. Different letters in the same line represent Tukey test for significant differences in strains among different samples (*p* < 0.05). Tween-80 used as a negative control, PGSS as a negative control.

## Data Availability

Samples are available from the authors.

## Abbreviation

*Michelia shiluensis* (*M. shiluensis*); gas chromatography–mass spectrometry (GC-MS); *Michelia shiluensis* fresh leaf aqueous extract (MSFLAE); *Michelia shiluensis* dried leaf aqueous extract (MSDLAE); 2,4,6-tripyridyl-s-trizine(TPTZ); 2,2′-azino-bis(3-ethylbenzothiazoline-6-sulfonic acid (ABTS); 2,2′-azobis [2-methylpropanamidine] dihydrochloride (AAPH); 1,1-diphenyl-2-picrylhydrazyl (DPPH); sodium ascorbate (VC); 2,6-di-tert-butyl-p-cresol (BHT); penicillin G sodium salt (PGSS); *Escherichia coli* (*E. coli*); *Staphylococcus aureus* (*S. aureus*); *Candida albicans* (*C. albicans*).
